# Evaluating the Feasibility of Machine-Learning-Based Predictive Models for Precancerous Cervical Lesions in Patients Referred for Colposcopy

**DOI:** 10.3390/diagnostics12123066

**Published:** 2022-12-06

**Authors:** Mingyang Chen, Jiaxu Wang, Peng Xue, Qing Li, Yu Jiang, Youlin Qiao

**Affiliations:** 1 School of Population Medicine and Public Health, Chinese Academy of Medical Sciences and Peking Union Medical College, Beijing 100005, China; 2 Diagnosis and Treatment for Cervical Lesions Center, Shenzhen Maternity & Child Healthcare Hospital, Shenzhen 518028, China

**Keywords:** machine learning, cervical precancers, colposcopy, predictive model

## Abstract

**Background:** Colposcopy plays an essential role in cervical cancer control, but its performance remains unsatisfactory. This study evaluates the feasibility of machine learning (ML) models for predicting high-grade squamous intraepithelial lesions or worse (HSIL+) in patients referred for colposcopy by combining colposcopic findings with demographic and screening results. **Methods:** In total, 7485 patients who underwent colposcopy examination in seven hospitals in mainland China were used to train, internally validate, and externally validate six commonly used ML models, including logistic regression, decision tree, naïve bayes, support vector machine, random forest, and extreme gradient boosting. Nine variables, including age, gravidity, parity, menopause status, cytological results, high-risk human papillomavirus (HR-HPV) infection type, HR-HPV multi-infection, transformation zone (TZ) type, and colposcopic impression, were used for model construction. **Results:** Colposcopic impression, HR-HPV results, and cytology results were the top three variables that determined model performance among all included variables. In the internal validation set, six ML models that integrated demographics, screening results, and colposcopic impression showed significant improvements in the area under the curve (AUC) (0.067 to 0.099) and sensitivity (11.55% to 14.88%) compared with colposcopists. Greater increases in AUC (0.087 to 0.119) and sensitivity (17.17% to 22.08%) were observed in the six models with the external validation set. **Conclusions:** By incorporating demographics, screening results, and colposcopic impressions, ML improved the AUC and sensitivity for detecting HSIL+ in patients referred for colposcopy. Such models could transform the subjective experience into objective judgments to help clinicians make decisions at the time of colposcopy examinations.

## 1. Introduction

Although cervical cancer is a preventable disease, it remains one of the most common cancers and causes of cancer-related death among women worldwide [1]. Cervical cancer also reflects global inequities, as its incidence and mortality rates in low- and middle-income countries (LMICs) are nearly twice and three times those in high-income countries, respectively. The World Health Organization (WHO) set a target that by 2030, 70% of women will be screened with a high-performance test by 35 years of age and again by 45 years of age [2]. Colposcopy-guided biopsy is crucial for detecting cervical precancers and determining treatment or further observation, but it is also the main bottleneck limiting screening performance. The diagnostic ability of many colposcopists is not favorable, as up to 40% of high-grade squamous intraepithelial lesions or worse (HSIL+) cases are missed in LMICs [3]. The consistency rate between colposcopic impressions and histopathological results varies widely, ranging from 52% to 66% [4,5,6]. As the screening modality gradually changes from cytology to primary HPV screening, cervical abnormalities are likely to be mild and difficult to identify, which aggravates the difficulty of the colposcopy diagnosis.

The American Society for Colposcopy and Cervical Pathology (ASCCP) has recommended modifying colposcopy practice on the basis of cytology, human papillomavirus (HPV) genotyping, and colposcopic impression [7,8]. However, the guidelines do not cover all situations. For instance, there are no uniform standards to determine follow-up or biopsy or direct treatment when facing non-16/18 high-risk HPV infections with mild cytological abnormalities. Colposcopists need to integrate more clinical information, such as age and reproductive history, to make such decisions, which inevitably depend on subjective experience. As women with a wide range of underlying precancer risks are referred for colposcopy every day [9], experience alone is not a long-term solution, especially for junior colposcopists. Thus, it is particularly important to establish an objective model that can predict the risk of HSIL+.

Recently, the application of machine learning (ML) methods to healthcare has been rapidly growing because of the increasing availability of large databases and computing power [10,11,12,13]. ML algorithms are often used to process multi-dimensional data, bringing new hope to solving clinical dilemmas. Previous ML studies that used screening results and demographics to predict cervical precancers performed well in screening scenarios [14,15,16,17]. However, there has been a lack of research into integrating colposcopic impressions with predicting HSIL+ among patients referred for coloscopies. Furthermore, relevant predictive models have always been developed on the basis of single-center data without external validation, which inevitably leads to model overfitting.

Therefore, this study integrates demographics, screening results, and colposcopic impressions to evaluate the feasibility of different ML models for predicting HSIL+ in patients referred for colposcopy. We aim to transform the subjective experience into objective predictive models for clinical use.

## 2. Materials and Methods

### 2.1. Study Design and Population

This is a multicenter, retrospective diagnostic study. The clinical records of patients who underwent colposcopic examination at seven hospitals in mainland China between January 2019 and October 2021 were collected. Women referred for colposcopy owing to abnormal screening results, clinical symptoms, or concerns about their own health were included, and those aged 24 to 65 with histopathological results were selected for further analysis. Demographic and clinical data were obtained, including age, gravidity, parity, menopause status, cytological results, HPV results, transformation zone (TZ) type, colposcopic impression, and histological diagnosis. 

This study was approved by the Institutional Review Board of the Chinese Academy of Medical Sciences and Peking Union Medical College (No. CAMS and PUMC-IEC-2022-022). Informed consent was waived owing to the retrospective nature of this study and the anonymization of patient information.

### 2.2. Screening Tests, Colposcopy, and Histology Diagnosis

Cytology results were classified into five categories in accordance with the Bethesda system, including negative for intraepithelial lesion or malignancy (NILM), atypical squamous cells of undetermined significance (ASC-US), low-grade squamous intraepithelial lesion (LSIL), atypical squamous cells—cannot exclude high-grade squamous intraepithelial lesion (ASC-H), and HSIL+. HPV results were categorized as HPV 16/18 positive, other HR-HPV positive, and HR-HPV negative. 

Experienced colposcopists assessed the type of TZ and gave a colposcopic impression (normal, LSIL, HSIL, or cancer) following the 2011 IFCPC colposcopic terminology for the cervix [18]. All abnormalities were biopsied, and endocervical curettage was performed if necessary. Histological diagnosis performed by experienced pathologists from local hospitals was considered the gold standard. Histological results were classified as normal, LSIL, and HSIL+ in accordance with the Lower Anogenital Squamous Terminology (LAST) system. Any disagreements were resolved by discussion. The worst grade of the dysplasia present was taken as the final diagnosis. 

### 2.3. Development and Validation of ML Models

Features were selected on the basis of literature reviews and expert discussion. Age, gravidity, and parity have been identified as risk factors for cervical cancer in previous studies and were included in our ML models [19,20]. The ASCCP guidelines recommend that colposcopy practice should be performed on the basis of cytology, HPV results, and colposcopic impression [7]. Thus, these variables were also used for model construction. Although the influence of menopause status and TZ type on the development of HSIL+ is not consistent, these variables play important roles in colposcopy practice and are recommended by experts to be included in the model [6,21,22]. 

The complete data were divided into internal and external sets on the basis of hospital site. Individuals collected from five hospitals were used as the internal set. The synthetic minority oversampling technique (SMOTE) was adopted to eliminate the impact of imbalanced data. In the oversampling process, the number of minority samples was randomly filled to the same number as that of the majority samples. The internal set was then randomly divided into the training and internal validation sets at a ratio of 8:2. The other two hospitals were used as the external validation set to assess the generalization of the model. We also used five-fold cross-validation to test the accuracy of models on the whole dataset from seven hospitals.

Six commonly used ML models were selected in this study, including logistic regression (LR), decision tree (DT), naïve bayes (NB), support vector machine (SVM), random forest (RF), and extreme gradient boosting (XGBoost). As a classical statistical model, the LR model performs well in a variety of statistical prediction and regression scenarios. The DT model divides the search space into smaller parts and searches for each part by asking yes or no questions. The NB model, based on the Bayesian inference theorem, can demonstrate the importance of different variables in the prediction process from another perspective. SVM with a radial basis function (RBF) maps input variables into a nonlinear hyperplane with higher feature spaces and can classify both linear and nonlinear data. The RF is an ensemble learning algorithm based on bagging. It uses different random samples to train multiple decision trees and the voting method to obtain the final classification result. XGBoost is an efficient and extensible learning classifier that is based on boosting. The objective function of XGBoost is regularized, which is beneficial to control overfitting and further improve model performance. The development of these models was operated on a Jupyter Notebook in Anaconda based on python 3.8.5. We used the sk-learn.tree decision tree classifier function to develop the DT model. The NB model was developed using sk-learn.naive.bayes, and the SVM model was built using the sk-learn.svm.SVC function. The random forest model was established by the RandomForestClassifier function in the sklearn.ensemble package. Additionally, the XGBoost model was developed through the XGBClassifier function in the XGBoost package. The model parameters were optimized by comparing the average accuracy in a five-fold cross-validation. The prediction function was used to generate the probability predicted by each model. We chose the optimal cut-off point, with excellent sensitivity and good specificity, for every model.

### 2.4. Statistical Analysis

Data analysis was performed using SAS version 9.4 (Cary, NC, USA). The chi-square test was used to compare differences in the distribution of variables between HSIL+ and <HSIL cases in the training and validation datasets. A two-sided *p* < 0.05 was regarded as statistically significant. Sensitivity, specificity, accuracy, the Matthews correlation coefficient (MCC), receiver operating characteristic (ROC) curves, and the area under the curve (AUC) were used to evaluate the diagnostic performance of different ML models and colposcopists at detecting HSIL+. The importance of variables in the SVM and NB models was calculated by estimating the decrease in AUC when the variable was removed from the model. The importance of variables in the LR, DT, RF, and XGBoost models was reflected by coefficients in the models. The overall-importance of the variable is obtained by averaging the variable ranking in six models.

## 3. Results

### 3.1. Characteristics of Study Population

**Figure 1:**
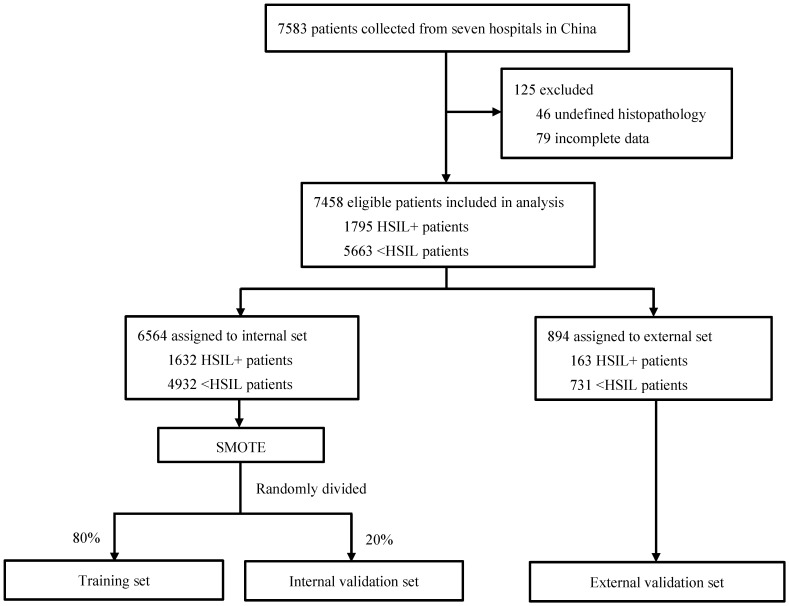
Flow chart showing the process of model development and validation. Abbreviations: <HSIL, normal or low-grade squamous intraepithelial lesion; HSIL+, high-grade squamous intraepithelial lesion or worse; SMOTE, synthetic minority oversampling technique.

Figure 1 shows the process of model development and validation. We identified 7583 patients who had undergone colposcopy during the study period, 125 of whom were excluded according to our predefined selection criteria. The data of 7485 patients were used to develop and validate the ML models. The training and internal validation set consisted of 1632 HSIL+ patients and 4932 <HSIL patients. The external validation set consisted of 163 HSIL+ patients and 731 <HSIL patients. Among the whole population, 4135 (55.4%) were aged between 30 and 45; most had one or two pregnancies (5079, 68.1%), one or two deliveries (6018, 80.7%), and no menopause (6287, 84.3%). There were 4073 (54.6%) patients with cytological ASC-US or above, 2479 (33.2%) with HPV 16/18 positivity, 3587 (48.1%) with other HR-HPV positivity; 38.2% (2013) of the women were infected by multiple HPV subtypes. In total, 2847 (38.2%) participants had type 3 TZ, while 3695 (49.5%) and 1629 (21.8%) had colposcopic impressions of LSIL and HSIL+, respectively. The characteristics of the study population in different datasets are displayed in Table 1. Compared with <HSIL controls, HSIL+ patients were more likely to be between 30 to 45 years old (*p* = 0.003), had more gravidities and parities, were more likely to be cytological LSIL+, HPV 16/18 positive, and infected by a single HR-HPV, had an HSIL+ colposcopic impression, and were less likely to have type 3 TZ (all *p* < 0.001).

### 3.2. Model Performance and Variable Ranking

Table 2 provides the predictive performance of each model and colposcopist for detecting HSIL+. In the internal validation set, the AUC, accuracy, sensitivity, specificity, and MCC for diagnosing HSIL+ by colposcopists were 0.830 (95% confidence interval (CI): 0.810–0.849), 83.27% (95% CI: 81.63–84.92%), 70.86% (95% CI: 67.99–73.74%), 95.06% (95% CI: 93.72–96.39%), and 0.695 (95% CI: 0.674–0.715), respectively. The six models that integrated demographics and screening results showed significant improvements in AUC (0.067 to 0.099) and sensitivity (11.55 to 14.88%). The DT model had a significantly higher AUC compared with the NB model (0.929 [95% CI: 0.918–0.940] vs. 0.897 [95% CI: 0.883–0.911]). The DT model was also slightly better than the other models in terms of accuracy, sensitivity, and MCC. 

**Figure 2:**
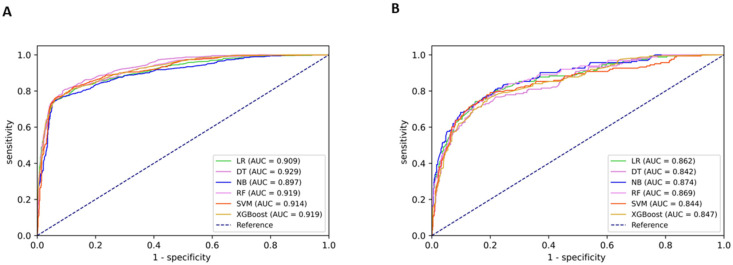
Performance of different models for detecting HSIL+. Displayed are receiver operating characteristic (ROC) curves and the area under the curves (AUC) of different models in detecting high-grade squamous intraepithelial lesions or worse (HSIL+). Different colors represent different models. LR, logistic regression; SVM, support vector machine; DT, decision tree; NB, naïve bayes; RF, random forest; XGBoost, extreme gradient boosting. (**A**) internal validation; (**B**) external validation.

In the external validation set, the AUC, accuracy, sensitivity, specificity, and MCC of colposcopists were 0.755 (95% CI: 0.708–0.803), 86.13% (95% CI: 83.86–88.40%), 58.90% (95% CI: 51.34–66.45%), 92.20% (95% CI: 90.26–94.15%), and 0.524 (95% CI: 0.491–0.557), respectively. Greater increases in AUC (0.087 to 0.119) and sensitivity (17.17 to 22.08%) were observed among the six models than in the internal validation set. In general, the performance of each model was similar but inferior to that in the internal validation set. Among them, the NB model showed a slightly higher AUC (0.874 [95% CI: 0.843–0.905]) than the other models, while the DT model performed relatively poorly for all indicators. The RF model yielded a relatively higher accuracy, specificity, and MCC but lower sensitivity. Figure 2 shows the ROC curves of the different models in the internal and external validation sets.

The combined confusion matrix and performance of the six models for detecting HSIL+ based on five-fold cross-validation are provided in Tables S1 and S2. The average accuracy, balanced accuracy, and MCC ranged from 86.56% to 89.13%, 80.94% to 83.48%, and 0.628 to 0.693, respectively.

The rankings of variable importance are shown in Table 3. Overall, colposcopic impression was the most important predictor, followed by HR-HPV infection and cytology. TZ type, HR-HPV multi-infection, and age ranked fourth, fifth, and sixth, respectively. Gravidity, parity, and menopause status all ranked seventh. Colposcopic impression had the strongest predictive effect in all six models. HR-HPV and cytology results ranked second or third in all models except for the LR model. TZ type was important in the LR, DT, and RF models but ranked lower in the SVM and XGBoost models. HR-HPV multi-infection ranked higher than fifth in the LR and XGBoost models. Age ranked higher than sixth in the SVM and NB models.

### 3.3. Subgroup Analysis

**Figure 3:**
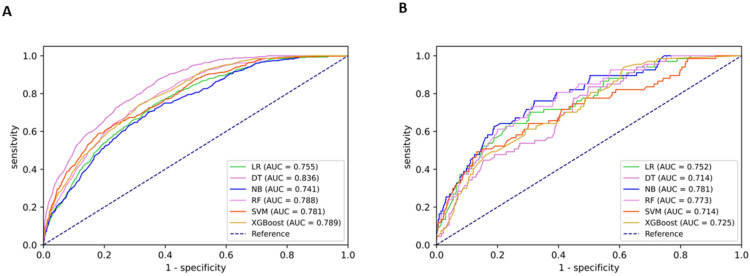
Performance of different models for detecting HSIL+ among patients diagnosed as <HSIL by colposcopists. Displayed are receiver operating characteristic (ROC) curves and the area under the curves (AUC) of different models in detecting histologically confirmed high-grade squamous intraepithelial lesions or worse (HSIL+) with colposcopic impression <HSIL. Different colors represent different models. LR, logistic regression; SVM, support vector machine; DT, decision tree; NB, naïve bayes; RF, random forest; XGBoost, extreme gradient boosting. (**A**) internal validation; (**B**) external validation.

Subgroup analysis was conducted to evaluate the predictive performance of different models in 487 HSIL+ patients who were diagnosed as <HSIL by colposcopists. As shown in Table 4, the DT model yielded the highest AUC (0.836 [95% CI: 0.819–0.854]) and sensitivity (87.38% [95% CI: 84.21–90.56%]) in the internal validation set, while it performed poorly in the external validation set, with an AUC of 0.714 (95% CI: 0.656–0.772) and sensitivity of 56.72% (95% CI: 44.85–68.58%). Although the NB model yielded relatively poor AUC (0.741 [95% CI: 0.718–0.764]) and sensitivity (74.29% [95% CI: 70.11–78.47%]) in the internal validation set, it performed the best in the external validation set, with an AUC of 0.781 (95% CI: 0.725–0.837) and sensitivity of 76.12% (95% CI: 65.91–86.33%). MCCs were low from 0.120 (95% CI: 0.096–0.143) to 0.277 (95% CI: 0.265–0.289) regardless of ML models and the validation set. Figure 3 shows the ROC curves of the six models in different datasets.

## 4. Discussion

In this study, we have developed and validated ML-based models to predict HSIL+ in patients referred to colposcopy. All six ML models that integrated demographics, screening results, and colposcopic impressions had significantly improved AUC and sensitivity compared with colposcopists, regardless of testing in the internal or external validation sets. Colposcopic impressions, HR-HPV results, and cytology results were the top three variables for determining model performance.

Colposcopy plays a vital role in detecting HSIL+, but its overall performance remains unsatisfactory. The sensitivity and specificity of colposcopistsfor detecting HSIL+ in our study were 70.86% and 95.06%, respectively, which were similar to the sensitivity and specificity of 71.6% and 98.0%, respectively, achieved by two expert colposcopists in a previous study [6]. However, the sensitivity was only 54.7% in another study carried out in China owing to various degrees of colposcopists’ clinical experience [23]. The primary task for colposcopists is to identify and treat high-grade diseases to reduce the risk of developing invasive cancer. However, several studies have shown that colposcopic diagnoses tend to underestimate rather than overestimate the pathology of biopsies [6,23,24]. This imprecision is partly because colposcopy largely depends upon the subjective experience of operators. Immaturity and the evolution of colposcopy diagnostic criteria also make junior colposcopists more confused [25]. Additionally, some potential lesions, visible at molecular and cellular levels, are not visible under colposcopy, which can be intensified with a change of screening modality from cytology to primary HPV screening. Therefore, more information, such as screening results and basic demographics, should be considered when determining follow-up, biopsy, or immediate treatment for patients referred for colposcopy.

Different ML models with various variables have been used for cervical cancer screening in previous studies. For example, Kahng et al. used an SVM model with age, cytology, and the presence of 15 HR-HPV genotypes to predict progression to cervical lesions, with an accuracy of 74.41% [14]. Karakitsos et al. developed a neural network classifier based on cytology, HPV, E6/E7 mRNA, and p16 immunostaining to identify cervical intraepithelial neoplasia grade 2+ (CIN2+), which obtained a significantly improved AUC (0.916) compared with cytological diagnosis alone (0.866) [16]. Another study reported that the valuable predictive factors were age, cytology, HR-HPV DNA/mRNA, E6 oncoprotein, HPV genotyping, and p16/Ki-67, which had an AUC of 0.92 for predicting CIN2+ [26]. Several studies have used epidemiologic risk factors and molecular markers to predict histologic grade or risk stratification on the basis of logistic regression results. For example, Rothberg et al. constructed a model using age, race, marital status, insurance, smoking history, income, and previous HPV results on around 100,000 women, obtaining an AUC of 0.81 for HSIL+ [17]. Another study used mRNA level, DNA index, parity, and age to achieve excellent discrimination for HSIL, with an AUC of 0.99 [15]. These studies further highlight the value of integrating clinical information and reflect the advantages of ML in processing multi-dimensional data. However, these studies were based on screening scenarios, and no model has been developed to further combine colposcopic impressions to predict HSIL+ under a diagnostic context.

All six ML models in our study showed improved AUC and sensitivity for detecting HSIL+ compared with colposcopists. However, the importance of colposcopy ranked first among all variables, no matter the model. This finding is consistent with previous research that showed that colposcopic impression had a stronger association with the final histological diagnosis of HSIL than pap smears or HPV 16/18 infections [27]. We also found that HR-HPV infection and cytology ranked second and third in overall variable importance, in line with colposcopy being a reason for referral, as proposed by the ASCCP [7]. In the 2017 ASCCP guidelines, serial cytology and HPV testing but not biopsy may be recommended when the risk of precancer is very low (no evidence of HPV 16/18, <HSIL cytology, and a completely normal colposcopic impression). In contrast, immediate treatment may be warranted for those at very high risk (at least two of the following: HPV 16/18 positive, HSIL cytology, and high-grade colposcopy impression). Silver et al. calculated pooled risk estimates for 32 strata on the basis of cytology, HPV 16/18 results, and colposcopy in a meta-analysis, and their results also support a risk-based approach to colposcopy and biopsy practice [28].

We found that TZ type and age ranked fourth and sixth among all variables, respectively. The proportion of HSIL+ in 30- to 45-year-olds was higher than in the other two age groups, which is consistent with the peak diagnosis age of HSIL being between 30 and 45 years [19]. The precise identification of the squamocolumnar junction (SCJ) and the evaluation of the TZ are crucial steps during colposcopy. In total, 38.2% of patients in our study had type 3 TZ. This proportion fluctuates greatly among different studied populations [6,29]. Previous studies have suggested that TZ type is associated with colposcopic accuracy, as higher accuracy and sensitivity for detecting HSIL+ were found in women with type 1/2 TZ compared with women with type 3 TZ [24]. In our study, the proportion of type 3 TZ in women with HSIL+ was less than that in women with <HSIL. This indicates that TZ type may not be directly related to developing HSIL+ but does affect the performance of colposcopy, which requires special attention in clinical practice to avoid the misdiagnosis of CINs.

Moreover, patients with increased gravidity or parity were more likely to have HSIL+. Previous studies have confirmed a direct relationship between reproductive factors and the risk of cervical cancer [20,30,31]. The ectopia of cervical columnar epithelium caused by delivery-related cervical traumas and high concentrations of estrogen and progesterone during pregnancy make the SCJ more susceptible to HPV infection. The hormonal profile and immunodepression caused by pregnancy may also favor infection or accelerate cervical carcinogenesis. Additionally, more pregnancies may represent insufficient condom use, and subsequently induced abortions may lead to joint infections of bacteria and viruses, which aggravates damage to the cervix. The impact of menopause status on the development of HSIL+ is complicated by hormone levels and immune status. Cervical atrophy, decreased cell detachment, and the contraction of the SCJ in postmenopausal women may result in the lower accuracy of colposcopy, which should be paid more attention to in clinical practice.

In total, 33.2% of HR-HPV-positive patients were infected with multiple HR-HPVs in our study, which is within the range of 26.3% and 43.2% that has been previously reported [32,33]. We found that a higher incidence of HSIL+ in patients with single infections than those with multiple infections. However, the association between multiple HPV infections and cervical lesions remains inconclusive. Some studies support multiple infections having no additive or synergistic effects on the development of HSIL+ [33,34], while other studies suggest that coinfection may act synergistically in cervical carcinogenesis [35]. Other data highlight the importance of the type of HPV infection. For example, a single infection with HPV 16 is more virulent than a multiple-infection pattern, while for HPVs 52, 53, 56, 51, 39, 66, 59, 68, and 35, a multiple-infection pattern is more likely to develop into HSIL+ than a single infection [32].

We found that the AUC of the DT model was significantly higher than that of the NB model in the internal validation set but that the NB model performed best in the external validation set despite no statistical significance being found. This finding is consistent with the algorithm, in which DT tends to overfit the training data, while NB is not prone to overfitting [36]. The DT is based on a hierarchical structure and has the advantage of good interpretability. However, it tends to overfit unless properly pruned, and it is unsuitable for all applications as it linearly separates samples. The NB model is easily implemented, highly scalable, and does not require many data entries to make proper classifications. Nevertheless, the independence assumption behind NB classifiers is quite bold and usually never holds in practice, leading to inaccuracies in the calculations of class probabilities. However, as long as the probability of the correct category is higher than those of the others, inaccuracies in the probability calculation will not affect outcomes. Both methods are fast and efficient, and in most cases, both will be tested before deciding. We also used two ensemble algorithms to construct the predictive model. RF is based on bagging, which fits many decision trees on different samples of the same dataset and averages the predictions. XGBoost is based on boosting, which adds ensemble members sequentially that correct the predictions made by prior models and outputs a weighted average of the predictions. These two models had no significant improvement in predicting HSIL+ compared to other base models in our study. 

To our knowledge, few studies have developed predictive models for cervical precancers in Chinese women referred for colposcopy. We also conducted internal and external validation of the ML models in a large set of samples from multiple centers. All variables we used are clinically easy to obtain. However, the study has some limitations that must be considered. First, it was a retrospective study, so prospective follow-up is needed to explore the long-term predictive performance of our models. Second, some variables that may improve the predictions of HSIL+, such as HPV infection history, smoking status, and sexual behavior, were not collected. Nevertheless, we have made the best use of the information available from existing sources. Third, in our study, colposcopy examinations were conducted by senior colposcopists, which may not be generalizable to all primary medical institutions. A way to better extrapolate the models using colposcopic information from junior colposcopists needs to be evaluated. As colposcopic impression was the variable with the greatest impact on model performance, improving the diagnostic ability of colposcopists is still a priority in the future. New technology such as artificial intelligence (AI) colposcopy and innovative forms of colposcopy training could bring exciting changes to the current situation [37,38,39]. Our predictive models trained by non-imaging clinical data could also be integrated with the current image-based AI colposcopy to make colposcopy practice more intelligent, objective, and comprehensive.

## 5. Conclusions

This study demonstrates that ML can improve the AUC and sensitivity for detecting HSIL+ in patients referred for colposcopy by incorporating demographics, screening results, and colposcopic impressions. These models can transform a subjective experience into an objective judgment to help clinicians make decisions at the time of colposcopy examinations.

## Figures and Tables

**Table 1:** Characteristics of the study population.

Variables	Total (*n* = 7458)			Training and Internal Validation Set (*n* = 6564)			External Validation Set (*n* = 894)		
	<HSIL †	HSIL+ ‡	*p*	<HSIL †	HSIL+ ‡	*p*	<HSIL †	HSIL+ ‡	*p*
Numbers	5663 (75.9)	1795 (24.1)		4932 (75.1)	1632 (24.9)		731 (81.8)	163 (18.2)	
Age			0.003			0.02			0.031
<30	984 (17.4)	251 (14.0)		850 (17.2)	233 (14.3)		134 (18.3)	18 (11.0)	
30–45	3099 (54.7)	1036 (57.7)		2761 (56.0)	945 (57.9)		338 (46.2)	91 (55.8)	
>45	1580 (27.9)	508 (28.3)		1321 (26.8)	454 (27.8)		259 (35.4)	54 (33.1)	
Gravidity			<0.001			<0.001			0.001
0	681 (12.0)	139 (7.7)		584 (11.8)	133 (8.1)		97 (13.3)	6 (3.7)	
1–2	3839 (67.8)	1240 (69.1)		3392 (68.8)	1137 (69.7)		447 (61.1)	103 (63.2)	
≥3	1143 (20.2)	416 (23.2)		956 (19.4)	362 (22.2)		187 (25.6)	54 (33.1)	
Parity			<0.001			<0.001			0.022
0	1029 (18.2)	246 (13.7)		892 (18.1)	226 (13.8)		137 (18.7)	20 (12.3)	
1–2	4525 (79.9)	1493 (83.2)		3946 (80.0)	1358 (83.2)		579 (79.2)	135 (82.8)	
≥3	109 (1.9)	56 (3.1)		94 (1.9)	48 (2.9)		15 (2.1)	8 (4.9)	
Menopause			0.321			0.422			0.863
No	4760 (84.1)	1527 (85.1)		4201 (85.2)	1404 (86.0)		559 (76.5)	123 (75.5)	
Yes	903 (15.9)	268 (14.9)		731 (14.8)	228 (14.0)		172 (23.5)	40 (24.5)	
Cytology			<0.001			<0.001			<0.001
NILM	2931 (51.8)	454 (25.3)		2418 (49.0)	400 (24.5)		513 (70.2)	54 (33.1)	
ASC-US	1660 (29.3)	396 (22.1)		1528 (31.0)	364 (22.3)		132 (18.1)	32 (19.6)	
LSIL	827 (14.6)	314 (17.5)		768 (15.6)	298 (18.3)		59 (8.1)	16 (9.8)	
ASC-H	113 (2.0)	189 (10.5)		96 (1.9)	164 (10.0)		17 (2.3)	25 (15.3)	
HSIL	132 (2.3)	442 (24.6)		122 (2.5)	406 (24.9)		10 (1.4)	36 (22.1)	
HR-HPV			<0.001			<0.001			<0.001
Negative	1311 (23.2)	81 (4.5)		1006 (20.4)	67 (4.1)		305 (41.7)	14 (8.6)	
Other HR-HPV positive	2906 (51.3)	681 (37.9)		2645 (53.6)	624 (38.2)		261 (35.7)	57 (35.0)	
HPV16/18 positive	1446 (25.5)	1033 (57.5)		1281 (26.0)	941 (57.7)		165 (22.6)	92 (56.4)	
HR-HPV multi-infection			<0.001			<0.001			<0.001
Negative	1311 (23.2)	81 (4.5)		1006 (20.4)	67 (4.1)		305 (41.7)	14 (8.6)	
Single infection	2864 (50.5)	1189 (66.3)		2476 (50.2)	1044 (64.0)		388 (53.1)	145 (88.9)	
Multiple infections	1488 (26.3)	525 (29.2)		1450 (29.4)	521 (31.9)		38 (5.2)	4 (2.5)	
Transformation zone			<0.001			<0.001			<0.001
Type 1	1646 (29.1)	557 (31.0)		1288 (26.1)	454 (27.8)		358 (49.0)	103 (63.2)	
Type 2	1730 (30.5)	678 (37.8)		1632 (33.1)	651 (39.9)		98 (13.4)	27 (16.6)	
Type 3	2287 (40.4)	560 (31.2)		2012 (40.8)	527 (32.3)		275 (37.6)	33 (20.2)	
Colposcopy			<0.001			<0.001			<0.001
Normal	2080 (36.7)	54 (3.0)		1775 (36.0)	40 (2.5)		305 (41.7)	14 (8.6)	
LSIL	3262 (57.6)	433 (24.1)		2893 (58.7)	380 (23.3)		369 (50.5)	53 (32.5)	
HSIL	318 (5.6)	1249 (69.6)		264 (5.4)	1172 (71.8)		54 (7.4)	77 (47.2)	
Cancer	3 (0.1)	59 (3.3)		0 (0.0)	40 (2.5)		3 (0.4)	19 (11.7)	

Category variables are presented as a number (percentage). † Histologically confirmed normal or low-grade squamous intraepithelial lesion. ‡ Histologically confirmed high-grade squamous intraepithelial lesion or worse. Abbreviations: NILM, negative for intraepithelial lesion or malignancy; ASC-US, atypical squamous cells of undetermined significance; LSIL, low-grade squamous intraepithelial lesion; ASC-H, atypical squamous cells—cannot exclude high-grade squamous intraepithelial lesions; HSIL, high-grade squamous intraepithelial lesion or worse; HR-HPV, high-risk human papillomavirus.

**Table 2:** Performance of different models and colposcopists for detecting HSIL+.

Model	AUC (95% CI)	Accuracy % (95% CI)	Sensitivity % (95% CI)	Specificity % (95% CI)	MCC (95% CI)
Internal validation set					
LR	0.909 (0.896–0.922)	82.31 (80.63–83.99)	83.87 (81.55–86.20)	80.83 (78.40–83.26)	0.647 (0.626–0.668)
SVM	0.914 (0.901–0.926)	83.83 (82.21–85.46)	83.66 (81.33–86.00)	83.99 (81.73–86.25)	0.677 (0.656–0.697)
DT	0.929 (0.918–0.940)	84.85 (83.26–86.43)	85.74 (83.53–87.95)	83.99 (81.73–86.25)	0.697 (0.677–0.717)
NB	0.897 (0.883–0.911)	81.65 (79.94–83.36)	83.35 (81.00–85.71)	80.04 (77.58–82.50)	0.634 (0.613–0.655)
RF	0.919 (0.907–0.931)	83.78 (82.15–85.41)	82.41 (80.01–84.82)	85.08 (82.88–87.27)	0.675 (0.655–0.696)
XGBoost	0.919 (0.907–0.930)	82.31 (80.63–83.99)	84.81 (82.54–87.08)	79.94 (77.47–82.41)	0.648 (0.627–0.669)
Colposcopists	0.830 (0.810–0.849)	83.27 (81.63–84.92)	70.86 (67.99–73.74)	95.06 (93.72–96.39)	0.695 (0.674–0.715)
External validation set					
LR	0.862 (0.830–0.895)	78.52 (75.83–81.22)	80.37 (74.27–86.47)	78.11 (75.11–81.11)	0.482 (0.449–0.515)
SVM	0.844 (0.807–0.882)	77.96 (75.25–80.68)	79.75 (73.59–85.92)	77.56 (74.54–80.59)	0.471 (0.438–0.504)
DT	0.842 (0.808–0.876)	74.38 (71.52–77.25)	77.91 (71.55–84.28)	73.60 (70.40–76.79)	0.415 (0.383–0.447)
NB	0.874 (0.843–0.905)	78.19 (75.48–80.90)	80.98 (74.96–87.01)	77.56 (74.54–80.59)	0.480 (0.448–0.513)
RF	0.869 (0.838–0.900)	80.87 (78.29–83.45)	76.07 (69.52–82.62)	81.94 (79.15–84.73)	0.496 (0.463–0.528)
XGBoost	0.847 (0.814–0.881)	77.85 (75.13–80.57)	77.91 (71.55–84.28)	77.84 (74.83–80.85)	0.460 (0.428–0.493)
Colposcopists	0.755 (0.708–0.803)	86.13 (83.86–88.40)	58.90 (51.34–66.45)	92.20 (90.26–94.15)	0.524 (0.491–0.557)

Abbreviations: HSIL+, high-grade squamous intraepithelial lesion or worse; AUC, area under the curve; MCC, Matthews correlation coefficient; LR, logistic regression; SVM, support vector machine; DT, decision tree; NB, naïve bayes; RF, random forest; XGBoost, extreme gradient boosting.

**Table 3:** Ranking of variable importance.

Model	Age	Gravidity	Parity	Menopause	Cytology	HR-HPV	HR-HPV Multi-Infection	Transformation Zone	Colposcopy
LR	8	9	7	6	5	2	3	4	1
SVM	4	7	5	6	2	3	8	9	1
DT	6	5	8	9	2	3	7	4	1
NB	4	6	7	8	2	3	9	5	1
RF	7	9	6	8	3	2	5	4	1
XGBoost	8	6	9	5	3	2	4	7	1
Overall †	6	7	7	7	3	2	5	4	1

The number 1 indicates that the variable ranks first in terms of importance, and 9 indicates that the variable ranks last among all variables. † The overall-importance of the variable is obtained by averaging the variable ranking in six models. Abbreviations: HR-HPV, high-risk human papillomavirus; LR, logistic regression; SVM, support vector machine; DT, decision tree; NB, naïve bayes; RF, random forest; XGBoost, extreme gradient boosting.

**Table 4:** Performance of different models for detecting HSIL+ among patients diagnosed as <HSIL by colposcopists.

Model	AUC (95% CI)	Accuracy % (95% CI)	Sensitivity % (95% CI)	Specificity % (95% CI)	MCC (95% CI)
Internal validation set					
LR	0.755 (0.733–0.777)	63.99 (62.67–65.31)	74.52 (70.36–78.69)	63.05 (61.66–64.43)	0.211 (0.200–0.222)
SVM	0.781 (0.759–0.803)	81.47 (80.40–82.53)	55.24 (50.48–59.99)	83.83 (82.77–84.88)	0.272 (0.260–0.284)
DT	0.836 (0.819–0.854)	64.33 (63.01–65.64)	87.38 (84.21–90.56)	62.25 (60.86–63.64)	0.277 (0.265–0.289)
NB	0.741 (0.718–0.764)	61.97 (60.64–63.30)	74.29 (70.11–78.47)	60.86 (59.46–62.26)	0.196 (0.185–0.207)
RF	0.788 (0.768–0.807)	76.53 (75.37–77.70)	61.90 (57.26–66.55)	77.85 (76.66–79.04)	0.251 (0.239–0.263)
XGBoost	0.789 (0.769–0.809)	73.98 (72.77–75.18)	63.81 (59.21–68.41)	74.89 (73.65–76.14)	0.236 (0.225–0.248)
External validation set					
LR	0.752 (0.693–0.812)	61.67 (58.17–65.17)	71.64 (60.85–82.43)	60.68 (56.99–64.37)	0.188 (0.160–0.216)
SVM	0.714 (0.644–0.784)	77.33 (74.31–80.34)	52.24 (40.28–64.20)	79.82 (76.79–82.85)	0.218 (0.189–0.248)
DT	0.714 (0.656–0.772)	62.89 (59.41–66.37)	56.72 (44.85–68.58)	63.50 (59.87–67.14)	0.120 (0.096–0.143)
NB	0.781 (0.725–0.837)	62.89 (59.41–66.37)	76.12 (65.91–86.33)	61.57 (57.90–65.25)	0.219 (0.189–0.249)
RF	0.773 (0.717–0.828)	75.71 (72.62–78.80)	61.19 (49.53–72.86)	77.15 (73.98–80.32)	0.250 (0.219–0.281)
XGBoost	0.725 (0.667–0.784)	63.97 (60.51–67.42)	64.18 (52.70–75.66)	63.95 (60.32–67.57)	0.166 (0.139–0.193)

Abbreviations: HSIL+, high-grade squamous intraepithelial lesion or worse; <HSIL, normal or low-grade squamous intraepithelial lesion; LR, logistic regression; SVM, support vector machine; DT, decision tree; NB, naïve bayes; RF, random forest; XGBoost, extreme gradient boosting; MCC, Matthews correlation coefficient.

## Data Availability

The datasets generated and/or analyzed during the current study are not publicly available because of personal information protection, patient privacy regulation, and medical institutional data regulatory policies but are available from the corresponding author upon reasonable request and with permission.

## Supplementary Materials

The following supporting information can be downloaded at: https://www.mdpi.com/article/10.3390/diagnostics12123066/s1. Table S1 Combined confusion matrix of different models based on five-fold cross-validation. Table S2 Performance of different models for detecting HSIL+ based on five-fold cross-validation.
