# Supplementary material for: Evaluating the Feasibility of Machine-Learning-Based Predictive Models for Precancerous Cervical Lesions in Patients Referred for Colposcopy
Source: Diagnostics (Basel). 2022 Dec 6;12(12):3066. doi: 10.3390/diagnostics12123066 (PMC9776471; doi:10.3390/diagnostics12123066)
Supplement: Supplementary file 1 [file diagnostics-12-03066-s001.zip › diagnostics-2041220-supplementary.pdf]

**Table S1 Combined confusion matrix of different models based on five-fold cross-validation**

| | Predicted <HSIL | Predicted HSIL+ |
|---|---|---|
| LR | | |
| Ture <HSIL | 5358 | 305 |
| True HSIL+ | 524 | 1271 |
| SVM | | |
| Ture <HSIL | 5344 | 319 |
| True HSIL+ | 492 | 1303 |
| DT | | |
| Ture <HSIL | 5314 | 349 |
| True HSIL+ | 548 | 1247 |
| NB | | |
| Ture <HSIL | 5198 | 465 |
| True HSIL+ | 537 | 1258 |
| RF | | |
| Ture <HSIL | 5334 | 329 |
| True HSIL+ | 495 | 1300 |
| XGBoost | | |
| Ture <HSIL | 5342 | 321 |
| True HSIL+ | 500 | 1295 |

Abbreviations: <HSIL, normal or low grade squamous intraepithelial lesion; HSIL+, high grade squamous intraepithelial lesion or worse; LR, logistic regression; SVM, support vector machine; DT, decision tree; NB, Naïve Bayes; RF, random forest; XGBoost, extreme gradient boosting

**Table S2 Performance of different models for detecting HSIL+ based on five-fold cross-validation**

| Model | Accuracy (%) | Balanced Accuracy (%) | MCC |
|---|---|---|---|
| LR | 88.88 ± 0.81 | 82.71 ± 0.98 | 0.685 ± 0.005 |
| SVM | 89.13 ± 0.81 | 83.48 ± 0.96 | 0.693 ± 0.005 |
| DT | 87.85 ± 0.85 | 81.50 ± 1.01 | 0.660 ± 0.006 |
| NB | 86.56 ± 0.88 | 80.94 ± 1.02 | 0.628 ± 0.006 |
| RF | 88.95 ± 0.81 | 83.31 ± 0.97 | 0.689 ± 0.005 |
| XGB | 88.99 ± 0.81 | 83.24 ± 0.97 | 0.670 ± 0.005 |

Data are mean value ± standard deviation

Abbreviations: HSIL+, high grade squamous intraepithelial lesion or worse; LR, logistic regression; SVM, support vector machine; DT, decision tree; NB, Naïve Bayes; RF, random forest; XGBoost, extreme gradient boosting; MCC, Matthews correlation coefficient
